# Erratum for He et al., “The adaptive growth and mechanisms of *Klebsiella pneumoniae* under sucrose and glucose exposure”

**DOI:** 10.1128/spectrum.01896-26

**Published:** 2026-08-25

**Authors:** Yunhui He, Fangfang Liu, Congcong Li, Jiayan Wu, Kewei Fan, Zewen Wen, Duoyun Li, Zhijian Yu, Tieying Hou

## ERRATUM

Volume 13, no. 12, e01603-25, 2025, https://doi.org/10.1128/spectrum.01603-25. The following footnote to the byline stating “Jiayan Wu, Kewei Fan, Zewen Wen, Duoyun Li, Zhijian Yu, and Tieying Hou contributed equally to this article” should be deleted.

The correct byline footnote is as follows: “Yunhui He, Fangfang Liu, and Congcong Li contributed equally to this article. The author order was determined by drawing straws.”

