# Supplementary material for: The adaptive growth and mechanisms of Klebsiella pneumoniae under sucrose and glucose exposure
Source: Microbiol Spectr. 2025 Oct 16;13(12):e01603-25. doi: 10.1128/spectrum.01603-25 (PMC12671095; doi:10.1128/spectrum.01603-25)
Supplement: Supplemental tables and figures — Tables S1 to S3 and Figures S1 to S7. [file spectrum.01603-25-s0010.docx]

**TABLE S1.** Strains used in this study

| **Strains** | **Characteristics** | **Source** |
| --- | --- | --- |
| NTUH-K2044 | Hypervirulent *Klebsiella pneumoniae*, hvKP | National Taiwan University College of Medicine |
| EKP18 | ESBLs-KP | Inpatients of Shenzhen Nanshan People’s Hospital (Sputum specimen) |
| EKP19 | ESBLs-KP, hvKP |  |
| EKP54 | ESBLs-KP, ST327 |  |
| EKP72 | ESBLs-KP, ST2938 |  |
| EKP50 | ESBLs-KP, ST327 | Inpatients of Shenzhen Nanshan People’s Hospital (Urine specimen) |
| EKP108 | ESBLs-KP, ST45 |  |
| LBKPN77 | ESBLs-KP, ST11 | Inpatients of Shenzhen Nanshan People’s Hospital (Peripheral blood specimen) |
| LBKPN79 | ESBLs-KP, ST11 | Inpatients of Shenzhen Nanshan People’s Hospital (Catheterised blood specimen) |
| K2044-Δ*scrA* | K2044 *scrA* knockout strain | This work |
| K2044-Δ*scrY* | K2044 *scrY* knockout strain | This work |
| ATCC 25922 | Reference strain，*Escherichia coli* | This laboratory |

ST: Sequence typing.

**TABLE S2.** Primers used in this study

| **Primers** | **Sequence** |  |
| --- | --- | --- |
| K2044-ΔscrA-F | 5’-TTCGTGGAGCTAGGCCTCCAATACCTGGCGCTTCGC-3’ | Upstream  594bp |
| K2044-ΔscrA-R | 5’-GCTATACGAACGGTAGGATCCAGTACCCTCTAAATGCATGATG-3’ |  |
| K2044-ΔscrA-F | 5’-CATACATTATACGAACGGTACCGCGCCTGGGTGGTGTCGATG-3’ | Downstream  634bp |
| K2044-ΔscrA-R | 5’-CCAGCCACCATGGGAGCTCTGCCATCGGCATTCTCGG-3’ |  |
| K2044-ΔscrY-F | 5’-TTCGTGGAGCTAGGCCTCTGGCCCTCGCCGACHC-3’ | Upstream  594bp |
| K2044-ΔscrY-R | 5’-CTATACGAACGGTAGGATCCGTTGGTGACATCCAAAGG-3’ |  |
| K2044-ΔscrY-F | 5’-CATACATTATACGAACGGTACCGGCAACCGGGGCGAC-3’ | Downstream  602bp |
| K2044-ΔscrY-R | 5’-CCAGCCACCATGGGAGCTCCTGCACATATCCAGCATG-3’ |  |
| KanR-scrA-F | 5’-CATCATGCATTTAGAGGGTACTGGATCCTACCGTTCGTATAGC-3’ | 1292bp |
| KanR-scrA-R | 5’-CATCGACACCACCCAGGCGCGGTACCGTTCGTATAATGTATG-3’ |  |
| PPK-CXF | 5’-GCAGTTTCATTTGATGCTCG-3’ | 1326bp |
| Kan-YZR | 5’-GGAGCAAGGTGAGATGACAGGAG-3’ |  |

**TABLE S3.** Antimicrobial susceptibility breakpoints

| Antimicrobials | CLSI（mg/L） | | | EUCAST（mg/L） | | |
| --- | --- | --- | --- | --- | --- | --- |
|  | R | I | S | R | I | S |
| Gentamicin | ≧8 | 4 | ≤2 | >2 | - | ≤2 |
| Meropenem | ≧4 | 2 | ≤1 | >8 | - | ≤2 |
| Ceftriaxone | ≧4 | 2 | ≤1 | >2 | - | ≤1 |
| Eravacycline^a^ | - | - | - | >0.5 | - | ≤0.5 |

a: Neither CLSI nor EUCAST currently recommend an antimicrobial drug susceptibility breakpoint for eclacycline against *K.pneumoniae.* Refer to EUCAST eravacycline antimicrobial drug susceptibility points for *Escherichia coli* for interpretation.


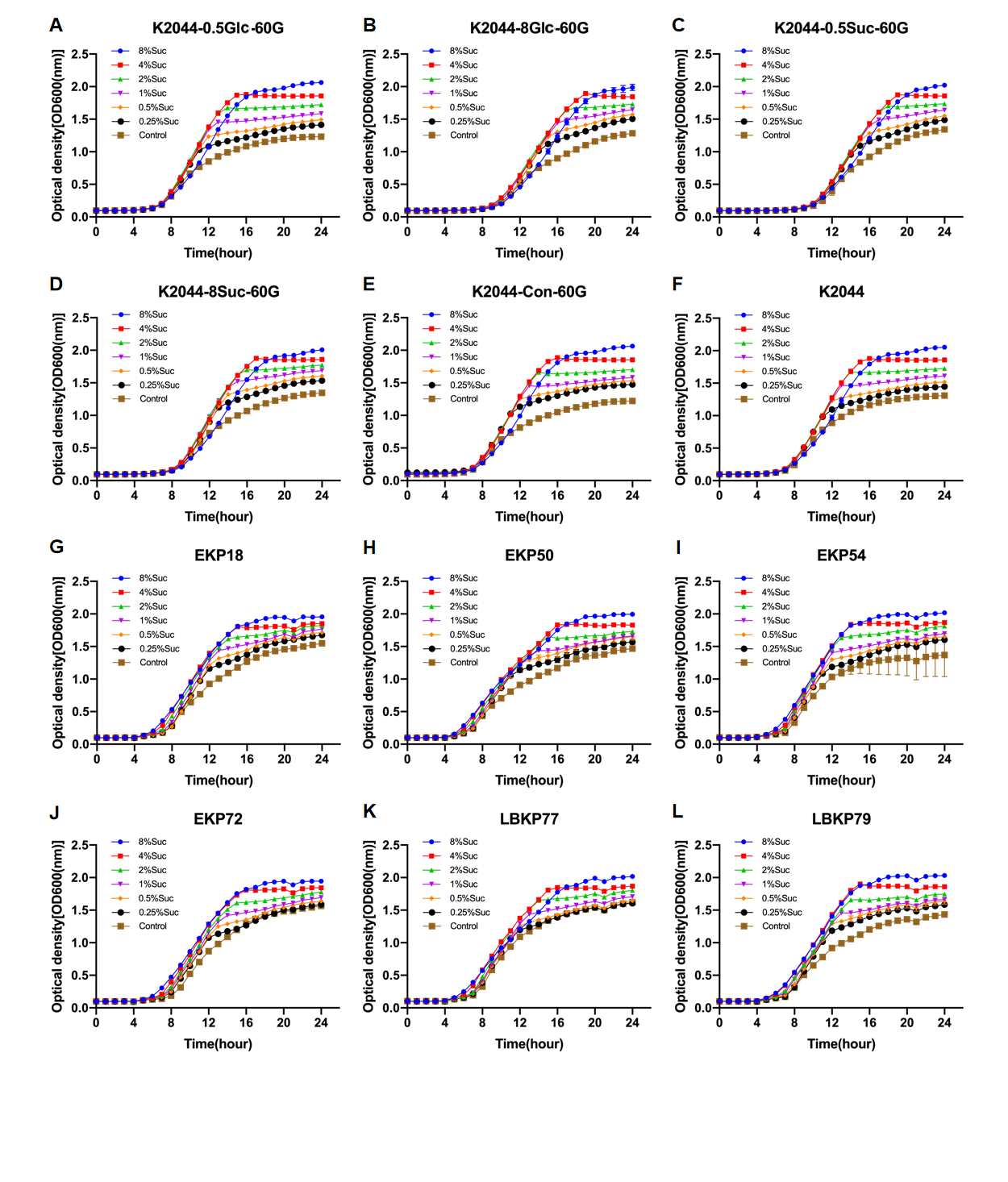


**FIG S1.** Growth curves of sugar-induced K2044 and other six clinical isolates at different initial sucrose concentrations. The induction strain of K2044 in 0.5% glucose MRS medium for 60 days of continuous passaging **(A)**, the induction strain of K2044 in 8% glucose MRS medium for 60 days of continuous passaging **(B)**, the induction strain of K2044 in 0.5% sucrose MRS medium for 60 days of continuous passaging **(C)**, the induction strain of K2044 in 8% sucrose MRS medium for 60 days of continuous passaging **(D)**, K2044 passaged strain in sugar-free MRS medium for 60 days of continuous passaging **(E)**, K2044 original strain **(F)**, respectively. Six *K. pneumoniae* clinical isolates **(G-L)**. Fig S1A-E is replotted data from Fig 2.

**
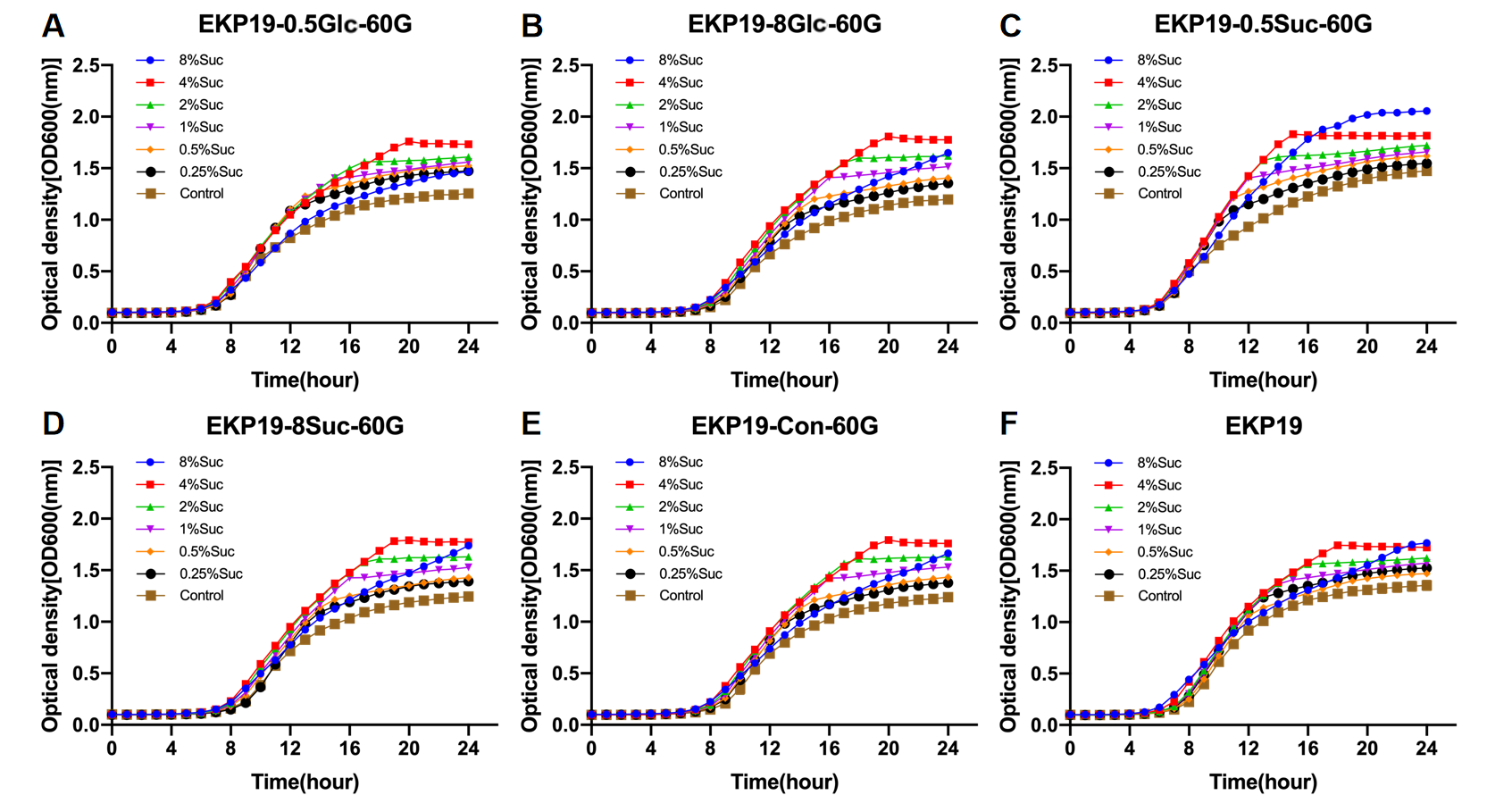
**

**FIG S2.** Growth curves of sugar-induced EKP19 at different initial sucrose concentrations. The induction strain of EKP19 in 0.5% glucose MRS medium for 60 days of continuous passaging **(A)**, the induction strain of EKP19 in 8% glucose MRS medium for 60 days of continuous passaging **(B)**, the induction strain of EKP19 in 0.5% sucrose MRS medium for 60 days of continuous passaging **(C)**, the induction strain of EKP19 in 8% sucrose MRS medium for 60 days of continuous passaging **(D)**, EKP19 passaged strain in sugar-free MRS medium for 60 days of continuous passaging **(E)**, EKP19 original strain **(F)**, respectively.

**
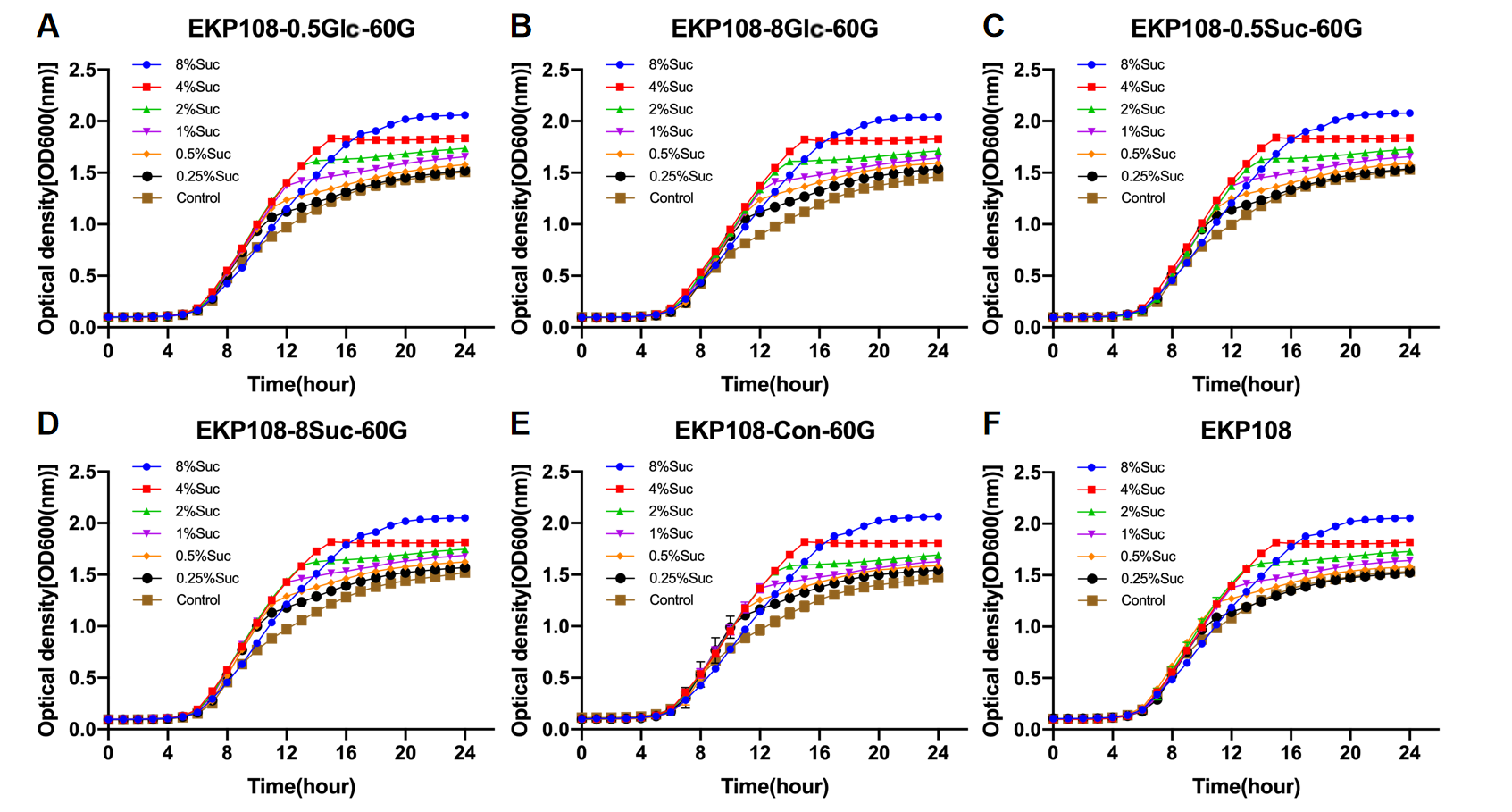
**

**FIG S3.** Growth curves of sugar-induced EKP108 at different initial sucrose concentrations. The induction strain of EKP108 in 0.5% glucose MRS medium for 60 days of continuous passaging **(A)**, the induction strain of EKP108 in 8% glucose MRS medium for 60 days of continuous passaging **(B)**, the induction strain of EKP108 in 0.5% sucrose MRS medium for 60 days of continuous passaging **(C)**, the induction strain of EKP108 in 8% sucrose MRS medium for 60 days of continuous passaging **(D)**, EKP108 passaged strain in sugar-free MRS medium for 60 days of continuous passaging **(E)**, EKP108 original strain **(F)**, respectively.

**
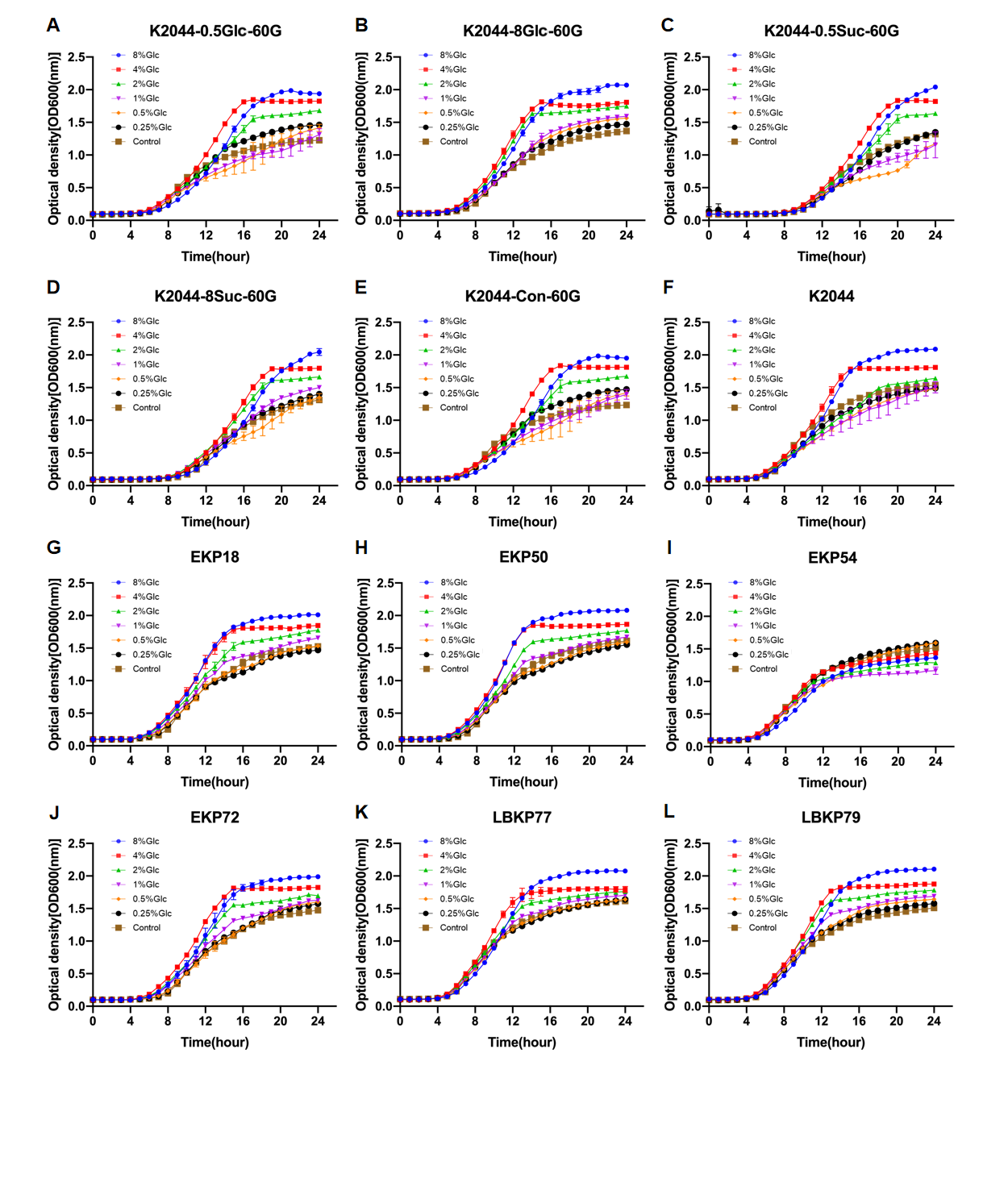
**

**FIG S4.** Growth curves of sugar-induced K2044 and other six clinical isolates at different initial glucose concentrations. The induction strain of K2044 in 0.5% glucose MRS medium for 60 days of continuous passaging **(A)**, the induction strain of K2044 in 8% glucose MRS medium for 60 days of continuous passaging **(B)**, the induction strain of K2044 in 0.5% sucrose MRS medium for 60 days of continuous passaging **(C)**, the induction strain of K2044 in 8% sucrose MRS medium for 60 days of continuous passaging **(D)**, K2044 passaged strain in sugar-free MRS medium for 60 days of continuous passaging **(E)**, K2044 original strain **(F)**, respectively. Six *K. pneumoniae* clinical isolates **(G-L)**.

**
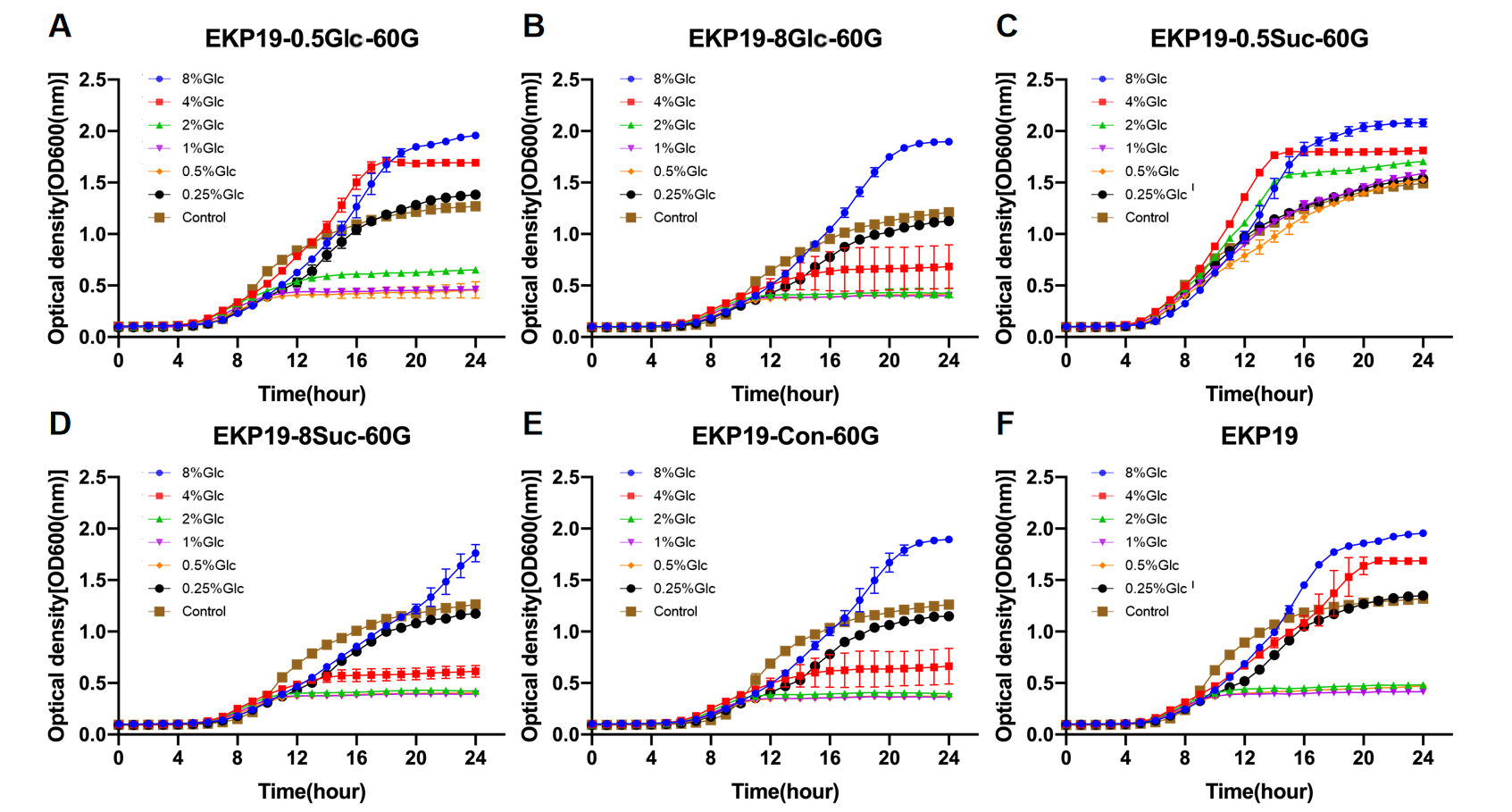
**

**FIG S5.** Growth curves of sugar-induced EKP19 at different initial glucose concentrations. The induction strain of EKP19 in 0.5% glucose MRS medium for 60 days of continuous passaging **(A)**, the induction strain of EKP19 in 8% glucose MRS medium for 60 days of continuous passaging **(B)**, the induction strain of EKP19 in 0.5% sucrose MRS medium for 60 days of continuous passaging **(C)**, the induction strain of EKP19 in 8% sucrose MRS medium for 60 days of continuous passaging **(D)**, EKP19 passaged strain in sugar-free MRS medium for 60 days of continuous passaging **(E)**, EKP19 original strain **(F)**, respectively.


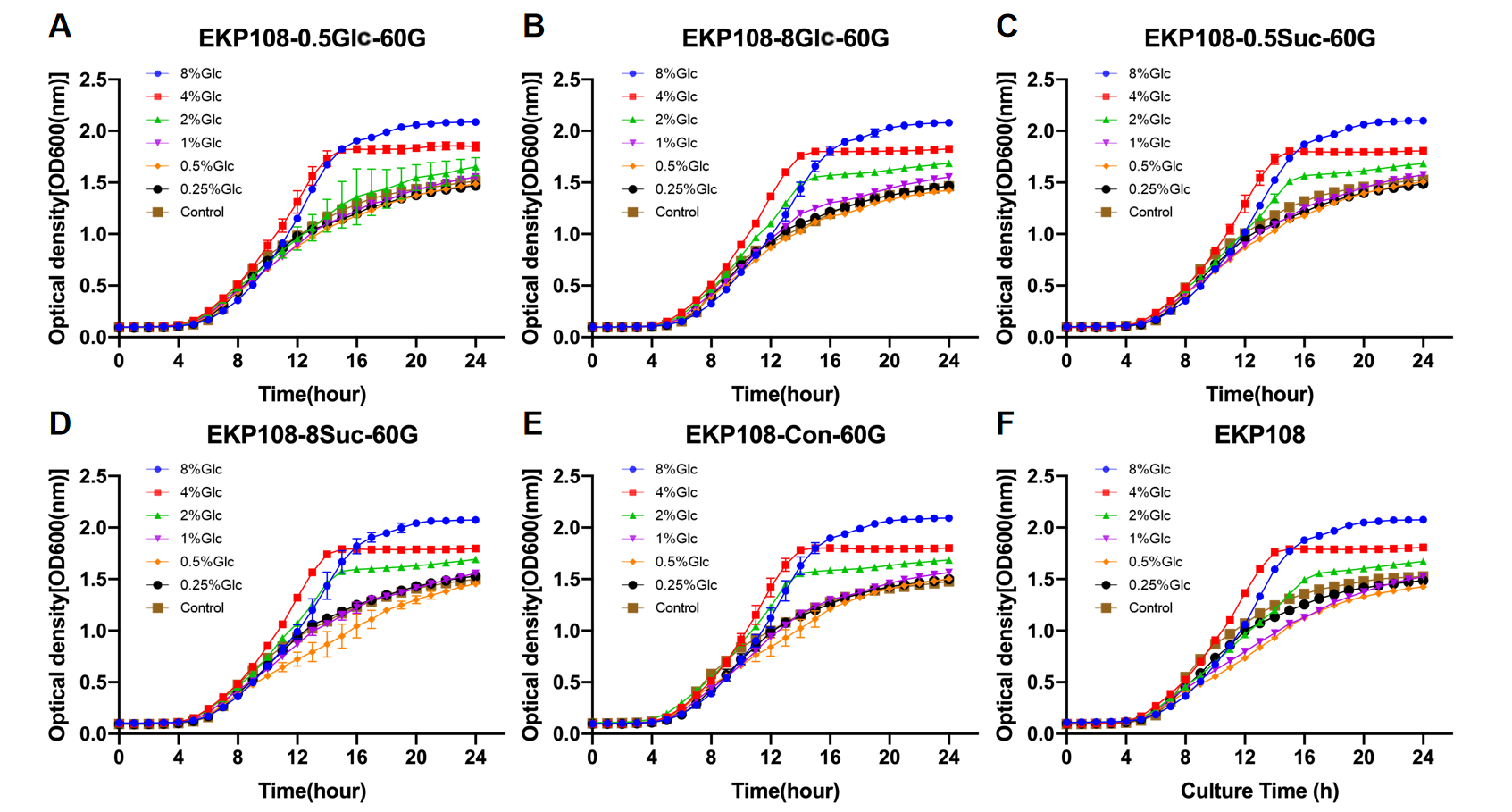


**FIG S6.** Growth curves of sugar-induced EKP108 at different initial glucose concentrations. The induction strain of EKP108 in 0.5% glucose MRS medium for 60 days of continuous passaging **(A)**, the induction strain of EKP108 in 8% glucose MRS medium for 60 days of continuous passaging **(B)**, the induction strain of EKP108 in 0.5% sucrose MRS medium for 60 days of continuous passaging **(C)**, the induction strain of EKP108 in 8% sucrose MRS medium for 60 days of continuous passaging **(D)**, EKP108 passaged strain in sugar-free MRS medium for 60 days of continuous passaging **(E)**, EKP108 original strain **(F)**, respectively.

**
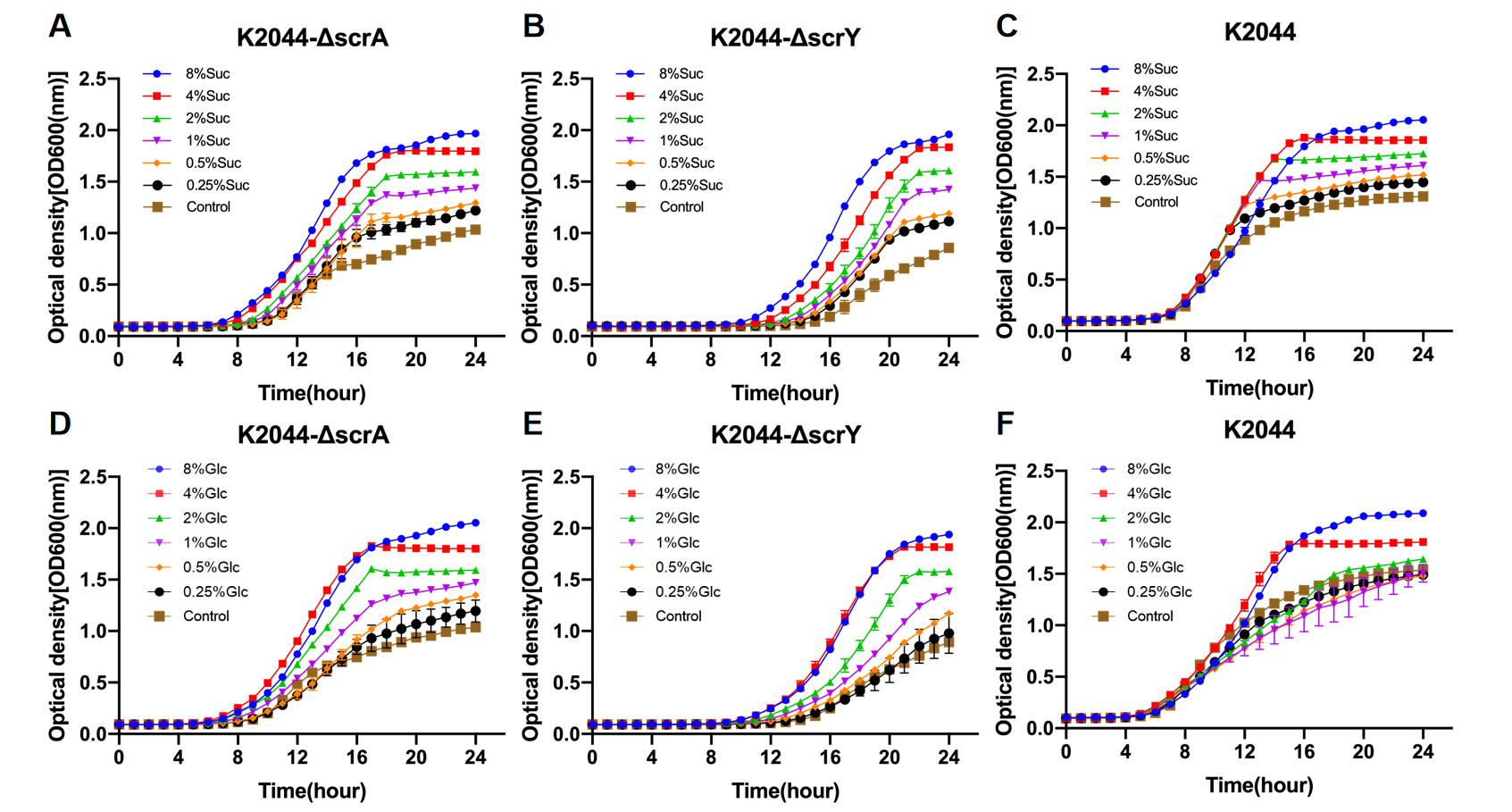
**

**FIG S7.** Growth curves of K2044-Δ*scrA* **(A, D)** and K2044-Δ*scrY* **(B, E)** at different initial sugar concentrations. K2044 WT **(C, F)**.
